# Edge Principal Components and Squash Clustering: Using the Special Structure of Phylogenetic Placement Data for Sample Comparison

**DOI:** 10.1371/journal.pone.0056859

**Published:** 2013-03-11

**Authors:** Frederick A. Matsen IV, Steven N. Evans

**Affiliations:** 1 Fred Hutchinson Cancer Research Center, Seattle, Washington, United States of America; 2 Department of Statistics, University of California, Berkeley, California, United States of America; American University in Cairo, Egypt

## Abstract

Principal components analysis (PCA) and hierarchical clustering are two of the most heavily used techniques for analyzing the differences between nucleic acid sequence samples taken from a given environment. They have led to many insights regarding the structure of microbial communities. We have developed two new complementary methods that leverage how this microbial community data sits on a phylogenetic tree. *Edge principal components analysis* enables the detection of important differences between samples that contain closely related taxa. Each principal component axis is a collection of signed weights on the edges of the phylogenetic tree, and these weights are easily visualized by a suitable thickening and coloring of the edges. *Squash clustering* outputs a (rooted) clustering tree in which each internal node corresponds to an appropriate “average” of the original samples at the leaves below the node. Moreover, the length of an edge is a suitably defined distance between the averaged samples associated with the two incident nodes, rather than the less interpretable average of distances produced by UPGMA, the most widely used hierarchical clustering method in this context. We present these methods and illustrate their use with data from the human microbiome.

## Introduction

Samples from microbial communities are complex, often containing millions of bacteria that differ to varying degrees. With high-throughput environmental sequencing, one can get a direct estimate of the composition of these microbial populations, even for microbes that cannot be cultured. Such estimates of composition can be too complex to compare directly, and so researchers have developed various ways of comparing populations. One option is to classify the collection of sequencing reads taxonomically, or group the reads into “operational taxonomic units” (OTUs) and then use a discrete comparison index such as the Jaccard index [1] to obtain a distance between samples. A shortcoming of such an approach is that it ignores the degree to which taxonomic labels represent similar or quite different organisms.

In 2005, Lozupone and Knight proposed a phylogenetics-based method to compute distances between samples that takes the natural hierarchical structure of the data into account. Their method, *unweighted UniFrac* [2], was followed by *weighted UniFrac* in 2007 [3] to incorporate abundance information. A key feature of both distances is that differences in community structure due to closely related organisms are weighted less heavily than differences arising from distantly related organisms. The UniFrac methodology can powerfully differentiate communities of interest in a variety of settings [4]–[6]; the papers describing the UniFrac variants have hundreds of citations as of the beginning of 2012. We have recently shown that the classical earth-mover's distance (a.k.a. Kantorovich-Rubinstein (KR) metric) [7] generalizes the weighted UniFrac distance.

Once distances have been computed between samples using UniFrac, these distances are typically fed into general-purpose ordination and clustering methods, such as principal coordinates analysis and UPGMA. Although it is appropriate to apply such techniques to distance matrices of this sort, the classical methods do not use the fact that the underlying distances were calculated in a specific manner, namely, on a phylogenetic tree. Consequently, in an application of principal components analysis, it is difficult to describe what the axes represent. Similarly, in hierarchical clustering, it is unclear what is driving a certain agglomeration step; although it can be explained in terms of an arithmetic operation, a certain amount of interpretability in the original phylogenetic setting is lost.

In this paper, we propose ordination and clustering procedures specifically designed for the comparison of microbial sequence samples that do take advantage of the underlying phylogenetic structure of the data. The input for these methods are collections of assignments of sequencing reads to locations on a “reference” phylogenetic tree: so-called *phylogenetic placements*. These placements may be obtained by software specialized to do model-based placement [8], [9], by using BLAST on a database built from the leaf sequences, or by clustering the sequences first and then building a tree on representative sequences as is commonly done for UniFrac.

Our *edge principal components analysis* (edge PCA) algorithm applies the standard principal components construction to a “data matrix” generated from the differences between proportions of phylogenetic placements on either side of each internal edge of the reference phylogenetic tree. Our *squash clustering* algorithm is hierarchical clustering with a novel way of merging clusters that incorporates information concerning how the data sit on the reference phylogenetic tree.

**Figure 1:**
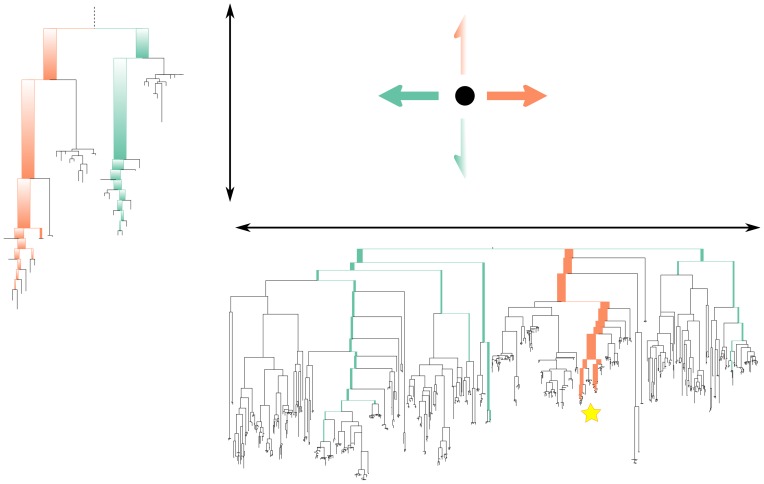
A graphical representation of the operation of edge principal components analysis (edge PCA). The phylogenetic distribution of reads for a given sample determines its position in the principal components projection. For the first axis, reads that fall below edges with positive coefficients on that axis' tree (marked in orange on the tree) move the corresponding sample point to the right, while reads that land on edges with negative coefficients (marked in green on the tree) move the corresponding sample point to the left. The second axis is labeled with a subtree of the first tree (the position of which is marked with a star on the first principal component tree): reads below edges with positive coefficients move sample points up, while reads below edges with negative coefficients move sample points down. The principal components shown here are the actual principal components for the example shown in Figures 4, 5, and 6.

**Figure 2:**
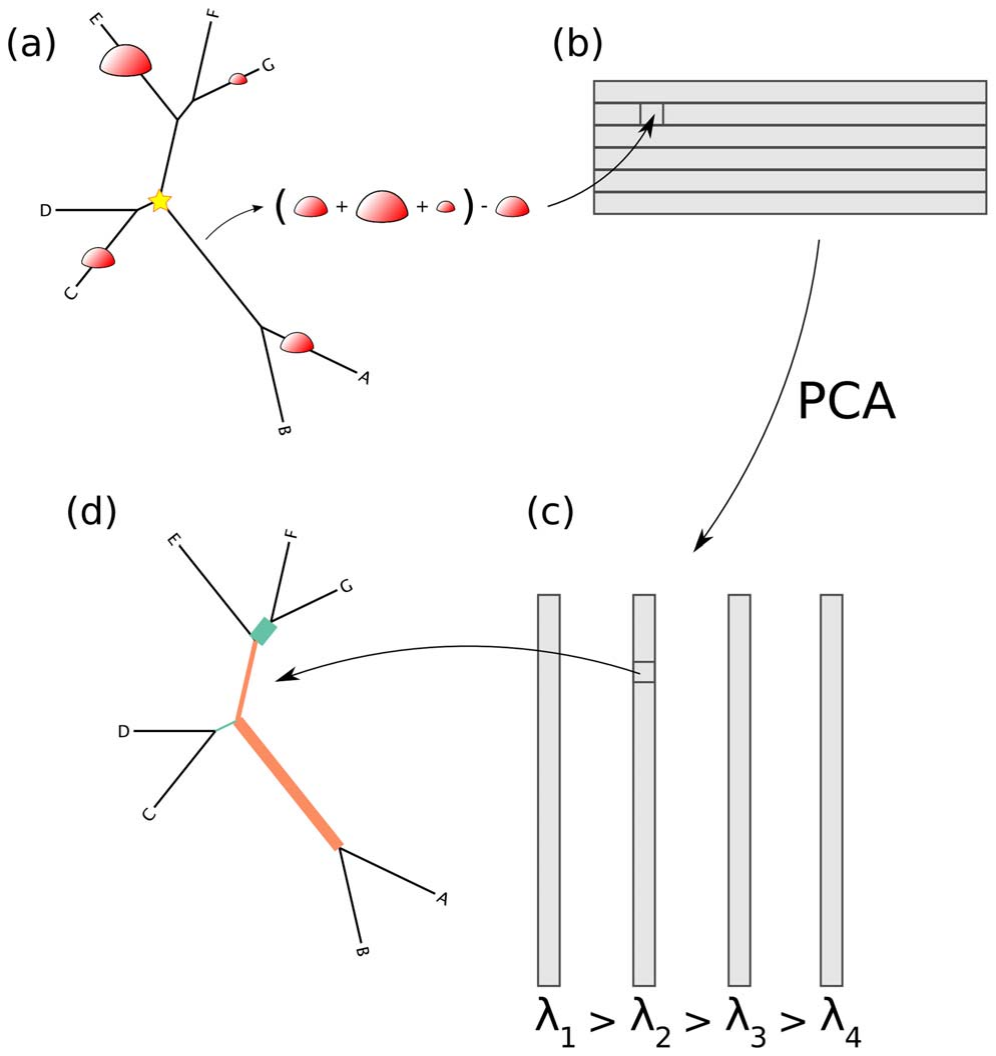
A visual depiction of the squash clustering algorithm. When two clusters are merged, their mass distributions are combined according to a weighted average. The edges of the reference tree in this figure are thickened in proportion to the mass distribution (for simplicity, just a subtree of the reference tree is shown here). In this example, the lower mass distribution is an equal-proportion average of the upper two mass distributions. Similarities between mass distributions, such as the similarity seen between the two clusters for the *G. vaginalis* clade shown here, are what cause clusters to be merged. Such similarities between internal nodes can be visualized for the squash clustering algorithm; the software implementation produces such a visualization for every internal node of the clustering tree. Note that in this figure only the number of reads placed on each edge is shown, although each placement has an associated location on each edge when performing computation.

The results of the analyses can be readily visualized and understood. The principal component axes of edge PCA can be pictured directly in terms of the reference phylogenetic tree, thereby attaching a clear interpretation to the position of a data point along that axis (Fig. 1). Edge PCA is also capable of picking up minor — but consistent — differences in collections of placements between samples: a feature that is important in our example application. The squash hierarchical clustering method is such that each vertex of the clustering tree is associated with a specific distribution of mass on the phylogenetic tree; the length of an edge in the clustering tree has a simple interpretation as the distance between the mass distributions associated with the two incident vertices (Fig. 2).

Edge PCA provides complementary information to a more traditional application of PCoA or NMDS to a distance matrix derived from UniFrac. Indeed, PCoA/NMDS gives a overall picture of how the biological samples compare in terms of overall similarities and differences, whereas edge PCA selects specific lineages that are high variance and compares the samples on that basis. This difference can be seen clearly in our example application.

The work presented here is distinct from recent work on data analysis methods for sets of trees. PCA on sets of trees has been developed in two contexts. Wang and Marron [10] have developed PCA on unlabeled planar trees, while Nye [11] has developed a PCA for phylogenetic trees with branch lengths and leaf labels. Those methods have the trees themselves as underlying objects of study; edge PCA, in contrast, takes vectors of edge weights on a single tree as input.

The work presented here shares some intent with double principal components (DPCoA) analysis as applied to distributions of phylotypes on a phylogenetic tree [12], [13]. The idea of a DPCoA analysis is to perform a principal components analysis on the phylotype abundance table in a way that down-weights differences between species that are close to one another on the phylogenetic tree. As such, it is somewhat similar to doing multidimensional scaling or principal components on the pairwise distance matrix generated by a UniFrac/KR analysis. It differs from the methods presented here because it only uses the tree in the form of a pairwise distance matrix; consequently it cannot leverage the edge-by-edge structure of the tree.

There are also some connections between edge PCA and the statistical comparison features of MEGAN [14] and LEfSe [15] in that the structure of a tree is used as part of a comparative framework. Our method and these methods all highlight regions of the tree for which important differences exist between samples. However, MEGAN and LEfSe work in the setting where one is explicitly trying to find statistically meaningful differences between pre-labeled sets of samples. The edge PCA algorithm, on the other hand, is an exploratory technique that does not attempt to make a hypothesis-testing statistical statement.

The ordination and clustering methods presented here are implemented in the guppy binary as part of the pplacer package, available at http://matsen.fhcrc.org/pplacer/. The methods take the recently-standardized JSON format for phylogenetic placements [16] as input. A tutorial and demonstration applying these methods can be found at http://fhcrc.github.com/microbiome-demo/.

## Results

### Intuitive presentation of methods

Here we give a simple overview of the two methods presented in this paper. The starting point for the methods is a collection of mappings of sequences onto a phylogenetic tree. This may be done by clustering sequences and building a tree *de novo*, by assigning sequences to the leaves of the tree using BLAST, or by mapping sequences into edges of the tree using model-based “phylogenetic placement” methods.

#### Edge principal component analysis

**Figure 3:**
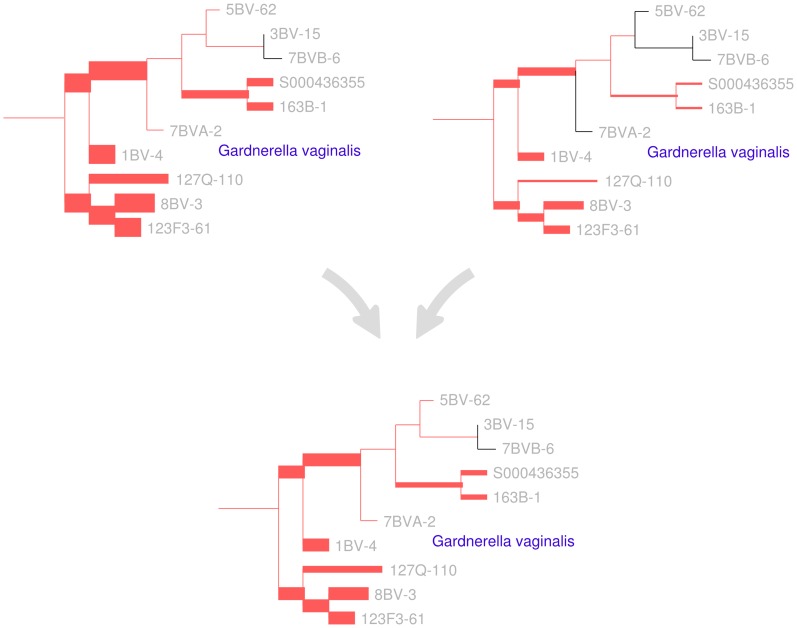
How the edge PCA algorithm works. (a) For every edge of the tree, the difference is taken between the number of reads on the non-root side the number of reads on the root side (root marked with a star). (b) The results of this are put into a matrix corresponding to the sample number (row) and the edge number (column). (c) The standard PCA algorithm is then applied, resulting in a collection of eigenvectors (the principal components) and eigenvalues. (d) These eigenvectors are indexed by the edges of the tree, and hence they can be mapped back onto the tree.

**Figure 4:**
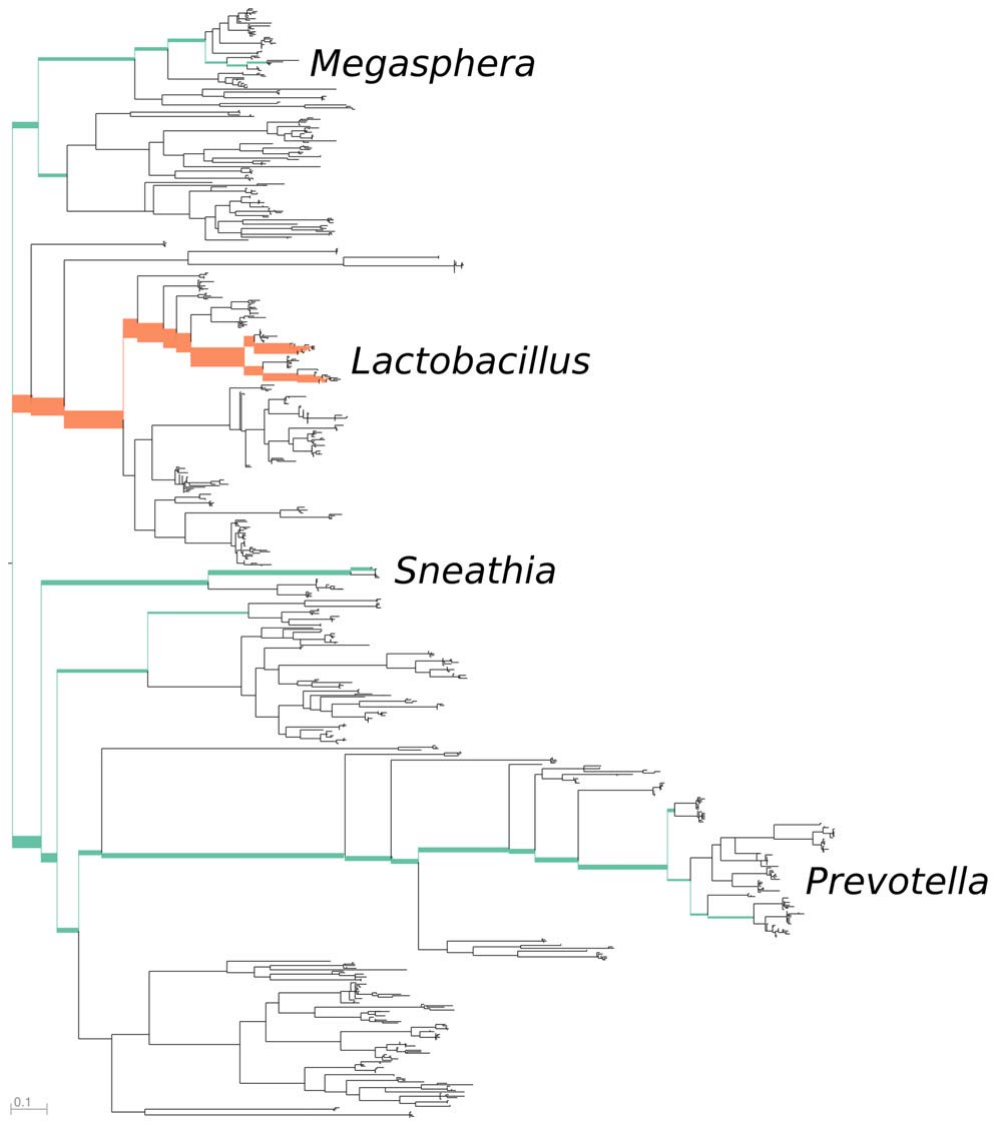
The first principal component for the combined vaginal data, representing about 56 percent of the variance. The reference tree is colored by principal component sign (positive colored orange, negative colored green) and thickened proportional to magnitude. The edges across which maximal between-sample heterogeneity is found are those leading to the *Lactobacillus* clade and those leading to the *Sneathia* and *Prevotella* clade. This axis corresponds to taxa that are important in the diagnosis of bacterial vaginosis, as *Sneathia* and *Prevotella* are associated with bacterial vaginosis, while *Lactobacillus* is associated with a healthy microbiome.

**Figure 5:**
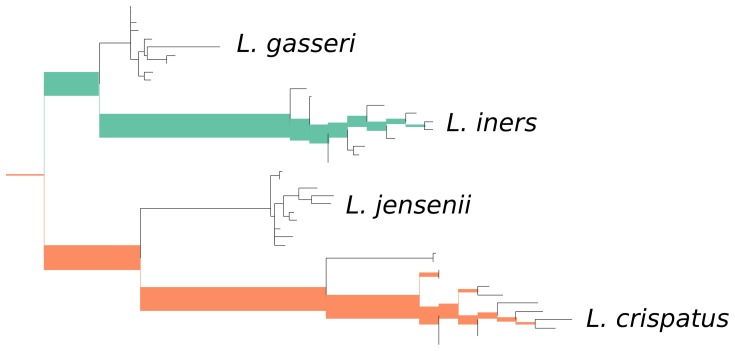
The second principal component for the combined vaginal data, representing about 24 percent of the variance. Low-weight regions of the tree are excluded from the figure. The edges across which maximal between-sample heterogeneity is found are those between two different *Lactobacillus* clades: *L. iners* and *L. crispatus*. Thus, the second important “axis” appears to correspond to the relative levels of these two species.

**Figure 6:**
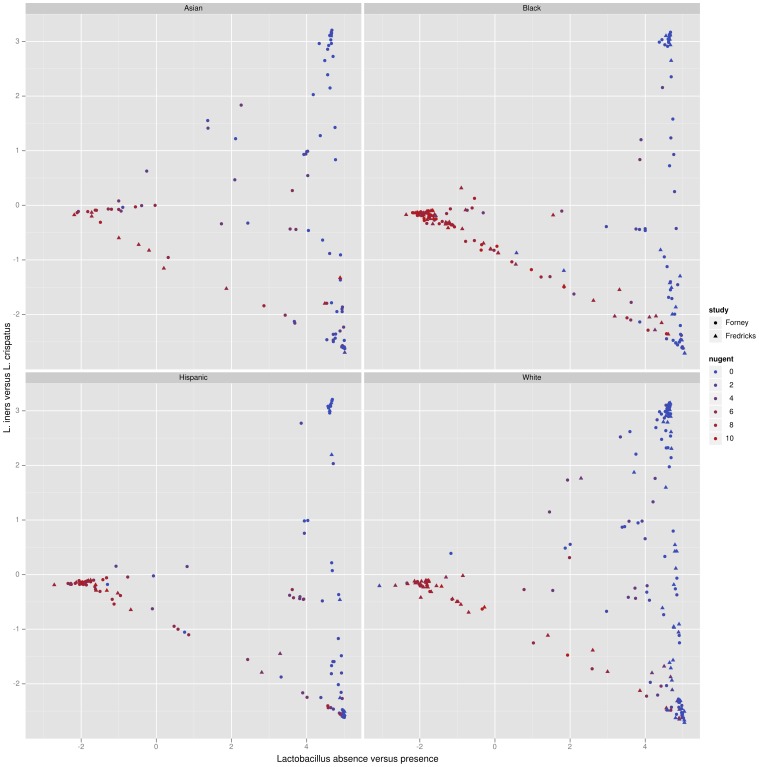
Edge principal components analysis (edge PCA) applied to the combined Forney and Fredricks data set and plotted separately. The axes for the edge principal components plot are described in Figures 4 (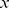-axis) and 5 (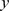-axis). The Nugent score is a diagnostic score for bacterial vaginosis, with high score indicating bacterial vaginosis.

Edge PCA is easily explained in the context of classical PCA, with the usual interpretation of PCA as a method to find a weighted sum of variables that maximizes variance. Edge PCA does a transformation such that the variables of interest are indexed by the edges of the tree, and these variables are then fed into the standard PCA machinery (Fig. 3). The consequent variable weightings can then be visualized on the tree (Fig. 4 and Fig. 5), and the samples can be plotted in the corresponding space (Fig. 6).

More intuitively, this process finds edges of the tree across which there is a high level of between-sample heterogeneity. That is, it finds those edges such that there are lots of reads on one side of the edge in a subset of the samples, and lots of reads on the other side of the edge in the complement of that subset. Those edges are then given a signed weight according to how strong this effect is. The sign of an edge considered in isolation is arbitrary, but the relative signs of any two edges indicate the extent of their anti-correlation in the between-sample heterogeneity. For example, if reads being mapped on the root side of one edge is significantly correlated with reads being mapped on the leaf-side of another edge, these edges will have different signs. The vector made in this manner, with the magnitudes of entries being determined by the level of between-sample heterogeneity, and the relative signs being determined by (anti-)correlations, is the first principal component vector. The second principal component is built in the same manner but after projecting out the first principal component, and so on.

In our visualization tool, each principal component eigenvector is represented by a single colored and thickened reference tree: the thickness of an edge is proportional to the magnitude of the corresponding entry of the eigenvector and the color specifies the sign of that entry (Fig. 4 and Fig. 5). For the trees shown here, orange signifies a positive entry, while green represents a negative entry.

Then, the projection of a given sample onto the plane is determined by the distribution of reads in the sample relative to the weighted edges. Specifically, a read on the leaf side of an edge with a positive weight will move the sample in the positive direction along that principal component, while a read on the root side will move it in a negative direction (Fig. 1). For edges with a negative weight the situation is reversed.

This behavior is achieved by a simple transformation of the data before applying the classical PCA machinery (Fig. 3). The first step is to build one vector per sample indexed by the edges of the tree filled by the “imbalance” between the fraction of reads on either side of that edge. This imbalance is defined, for a given edge 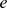 and sample 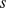, by cutting the tree in two by removing 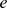 (and any associated placements), then taking the difference between the number of reads of 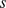 in the part of the tree containing the root minus the fraction in the part of the tree not containing the root. Edge PCA is then simply standard principal components analysis applied to the samples-by-edges data matrix created in this way. Namely, we construct the 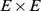 covariance matrix of this data matrix and then calculate its eigenvalues and their corresponding eigenvectors. Each eigenvector can be displayed on the tree, because the coordinates of the eigenvector correspond to internal edges of the tree. A large entry in an eigenvector corresponding to one of the bigger eigenvalues identifies an edge across which there is substantial heterogeneity among the associated set of mass differences (see Methods). Moreover, we can project each sample onto an eigenvector to visualize how the sample is spread out with respect to that “axis” (Fig. 1 and 6).

A significant emphasis of the edge PCA methodology is to obtain clearly interpretable axes for projection, and this is easiest when the eigenvectors have distinct sets of nonzero entries. When that is the case, a read in a certain region of the tree will move the corresponding sample point in one direction only. The *support* of a vector is the set of nonzero indices of that vector, thus the degree to which nonzero entries of principal components appear on shared edges will be called *support overlap*. We describe two means of support overlap minimization: one is rotating the principal component axes in the plane that they span, and the other is an explicit penalization scheme.

The rotation support overlap minimization simply rotates the principal components in the space that they span. For example, the rotation of the two principal components 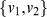 is 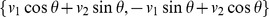. This rotation can greatly decrease the overlap of the support vectors, for example the pair of vectors 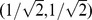 and 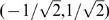 when rotated become 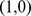 and 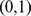. By rotating in the space spanned by the principal component eigenvectors, the projection of the points in the principal component space are correspondingly rotated, thus preserving the relative positions of the points in the principal component space. Although it preserves their relative positions, it does lose the original meaning of the principal component vectors: for example, the first dimension in this rotated space is no longer the component of maximal variance, although the proportion of the total variance in the subspace spanned by first 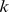 vectors is unchanged. Nevertheless, we have found this rotation to be useful for finding structure in edge PCA applications.

The second approach is to explicitly penalize the overlap between the second eigenvector and the first by subtracting out a measure of their overlap. As described in the Methods section, we have defined the second “penalized component” as having the second eigenvector 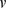 be chosen to maximize 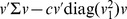 for some positive 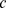, where 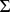 is the covariance matrix and 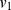 is the first eigenvector. However, we have not had tidy results using this explicit penalization, possibly because the first principal component is fixed and the second is then modified to avoid overlap with the first.

#### Squash clustering

Squash clustering is a type of hierarchical clustering that also uses the structure of the data to visualize what is happening with the clustering in more detail than is possible using a distance matrix only. The starting point is, as before, a collection of reads placed on a phylogenetic tree. Such a collection may be thought of as a distribution of a unit amount of mass across the tree. In the simplest setting, for a collection of 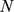 placements on a tree each read is given mass 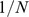; that mass is assigned to the “best” position for that read on the tree. Another option is to distribute the 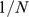 mass for a given read across the tree in proportion the posterior probability of assignment of that read to various positions (see Methods).

This mass distribution may be used to produce distances between collections of phylogenetic placements. Given two samples for a given locus, each sample is placed individually on the phylogenetic tree, and so each sample is thought of as a distribution of mass on the tree. The Kantorovich-Rubinstein (KR) or “earth-mover's” distance may then be used to quantify the difference between those two samples. This distance is defined rigorously in [7], but the idea is simple to explain. Imagine that the phylogenetic tree is a road network and that each sample is represented by the distribution of a unit of mass into piles of dirt along this road network. The distance between two samples is then defined to be the minimal amount of “work” required to move the dirt in the first configuration to that in the second configuration (in this context the amount of work needed to move an infinitesimal mass 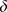 a distance 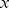 is defined to be 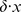). Thus, similar collections of phylogenetic placements result in similar dirt pile configurations that don't require much mass movement to transform one into the other, while quite different collections of placements require that significant amounts of mass must move long distances across the tree. This distance is classical, having roots in 18th century mathematics, and is a generalization of the *weighted UniFrac* distance [3], [7].

Squash clustering is hierarchical clustering using the KR distance but with a different way of using merged clusters: rather than taking averages of distances as is done in average-linkage clustering (also known as UPGMA), in squash clustering one takes distances between averages of samples. That is, given a collection of mass distributions on the reference phylogenetic tree, each of which correspond to a cluster that has been built at some stage of the procedure, when the procedure merges two clusters one simply takes a weighted average of the two corresponding mass distributions to get the mass distribution that corresponds to the new, larger cluster (Fig. 2). The “squash” terminology describes this averaging procedure: the original mass distributions for a given cluster are stacked on top of one another and then “squashed” down to produce a new object with unit total mass.

Every internal vertex of the clustering tree is associated with a distribution of mass on the phylogenetic tree, i.e. the squashed mass for the samples below that vertex. The length of an edge between two arbitrary adjacent vertices on the tree can be computed by using the KR distance between the distributions of mass corresponding to those vertices. This edge length calculation gives the resulting trees an appearance that differs from that of UPGMA trees because the lengths of the paths from the root to the various leaves are no longer all the same (i.e. the tree is typically not *ultrametric*).

The results of a squash clustering procedure are more transparent than the equivalent runs of other distance-based clustering procedures. Because of the merging process, each step of squash clustering operates on *exactly* the same type of mathematical object: a mass distribution on a phylogenetic tree. These mass distributions can be visualized, revealing the similarities that are driving a particular clustering step (Fig. 2).

In contrast, for UPGMA or other distance-based hierarchical clustering techniques, the internal nodes are represented by fundamentally different sorts of objects than the leaves. The internal nodes for the classical methods are represented by an agglomeration of points, and hierarchical clustering variants all have different ways of using the collection of between-point distances to compute distances between agglomerations of points. Consequently, it is not possible to find a manifestation of an internal node (like the equivalent of one of the mass distributions in Figure 2) where the distances to that manifestation are the distances used to create the clustering tree.

These internal node visualizations are automatically generated by the software implementation of the squash clustering algorithm. An example application of both edge PCA and squash clustering can found in our tutorial at http://fhcrc.github.com/microbiome-demo/.

### Example application: the vaginal microbiome

In this section we apply our clustering and ordination methods to pyrosequencing data from the vaginal microbiome. The “Fredricks” data set consists of sequence information from swabs taken from 242 women from the Public Health, Seattle and King County Sexually Transmitted Diseases Clinic between September 2006 and June 2010 of which 222 samples resulted in enough material to analyze [17] (Sequence Read Archive submission SRA051298). DNA was extracted and the 16S gene was amplified in the V3–V4 hypervariable region using broad-range primers and sequenced using a 454 sequencer with FLX chemistry. Sequences were pre-processed using the R/Bioconductor [18], [19] package *microbiome*. The “Forney” data set is an analogous data set of 454 reads from the V1–V2 hypervariable region amplified from vaginal swabs [20]. These sequences were downloaded as Sequence Read Archive submission SRA022855. The stability of reads from different regions of the same gene is the subject of a manuscript under preparation.

A custom maximum likelihood reference tree consisting of relevant sequences from RDP [21] and a local collection was built using *RAxML* 7.2.7 [22] using the GTR+4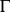 model as described in [17]. Sequences were aligned with Infernal v1.0.2 [23], and placed into this tree using pplacer [9] with the default parameter choices along with the -p and –inform-prior options.

The principal components for the vaginal samples independently recover previous knowledge about the contribution of certain microbial species to distinct types of vaginal microbial environment. A microscopic examination of Gram-stained specimens from the vaginal mucosa can be used to define a diagnostic criterion called the *Nugent score*. The Nugent score ranges from 0 to 10, with a high number indicating bacterial vaginosis (BV). The scoring criteria include a relative paucity of gram-positive rods described as *Lactobacillus* morphotypes, an abundance of bacteria resembling *Gardnerella* and *Bacteroides* species (small gram variable and gram negative rods, respectively), and an abundance of curved gram-negative rods [24]. The edge principal component algorithm appears to both agree with and extend these microscopic criteria: the first principal component for the vaginal data set identifies a negative association between *Lactobacillus* species versus species belonging to *Sneathia* and *Prevotella* (both gram-negative rods) and *Megasphera* (gram negative cocci) (Fig. 4). Both *Prevotella* and *Megasphera* have been independently identified as prevalent members of the vaginal microbiome, and are associated with a clinical diagnosis of BV [20], [25]. The second principal component reveals that important differences between samples exist at the species level. Indeed, it highlights the substantial amount of heterogeneity between the amount of two *Lactobacillus* species observed: *L. iners* and *L. crispatus* (Fig. 5). This latter observation is interesting from the medical perspective, as the Nugent criteria attribute the same significance to all *Lactobacillus* morphotypes regardless of species. In the context of the edge PCA analysis, however, a distinction is made between the two *Lactobacillus* species based on the population distribution of other organisms in the sample.

The samples from the two studies form a revealing pattern when plotted on these axes along with the corresponding Nugent score (Fig. 6). As described above, samples on the left side have *Sneathia* and *Prevotella* and lack *Lactobacillus* while those on the right side have the opposite. Samples on the bottom have lots of *L. iners* and a small amount of *L. crispatus*, while those on the top have the opposite.

*Lactobacillus* is associated with a low Nugent score and thus a negative BV diagnosis; in the results presented here *L. crispatus* dominated samples are not found to have a high Nugent score (indicating BV), while *L. iners* dominated samples sometimes are. In both the Forney and the Fredricks data sets, the samples with the highest Nugent score lie on a continuum of samples from the left to the lower right (from *Sneathia*/*Prevotella* to *L. iners* -dominant). A similar pattern is observed when the samples are divided by race (Fig. S2). Reviewing the taxonomically classified data from the Fredricks study confirms this trend. These plots indicate the possibility of a medically relevant difference between these two *Lactobacillus* species in a pattern that is consistent between two large, independent studies. It is also significant that phylogenetic placement on a reference tree containing full-length 16S rRNA gene sequences allows a direct comparison between the two data sets despite the fact that each sequenced a different region of the 16S rRNA gene. We emphasize that the PCA was **not** informed of either the Nugent score associated with the specimens or the taxonomic classifications of the sequences.

**Figure 7:**
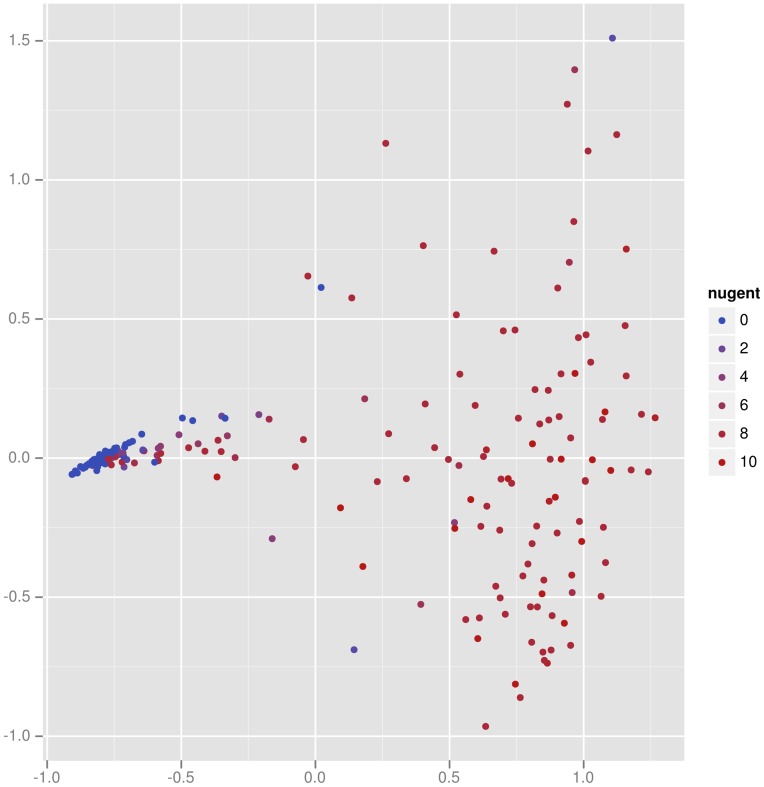
Principal coordinates analysis applied to the Fredricks vaginal data set.

Principal coordinates (PCoA) and multidimensional scaling (MDS) form a complementary set of techniques to edge principal components. PCoA applied in this context (Fig. 7) demonstrates two important facts about the vaginal specimens (a similar picture results from MDS; results not shown). First, it is clear that the BV negative (small Nugent score) specimens are very similar to one another in composition, and that the BV positive (high Nugent score) specimens are different from one another. This information is not recovered by the edge PCA analysis, which instead finds interesting structure within the BV negative specimens. This example emphasizes the complementary nature of edge PCA and these more classical methods, where the former gives specific information about the changes of the relative proportions of phylogenetic groups, whereas the latter gives a comparison of the overall composition.

**Figure 8:**
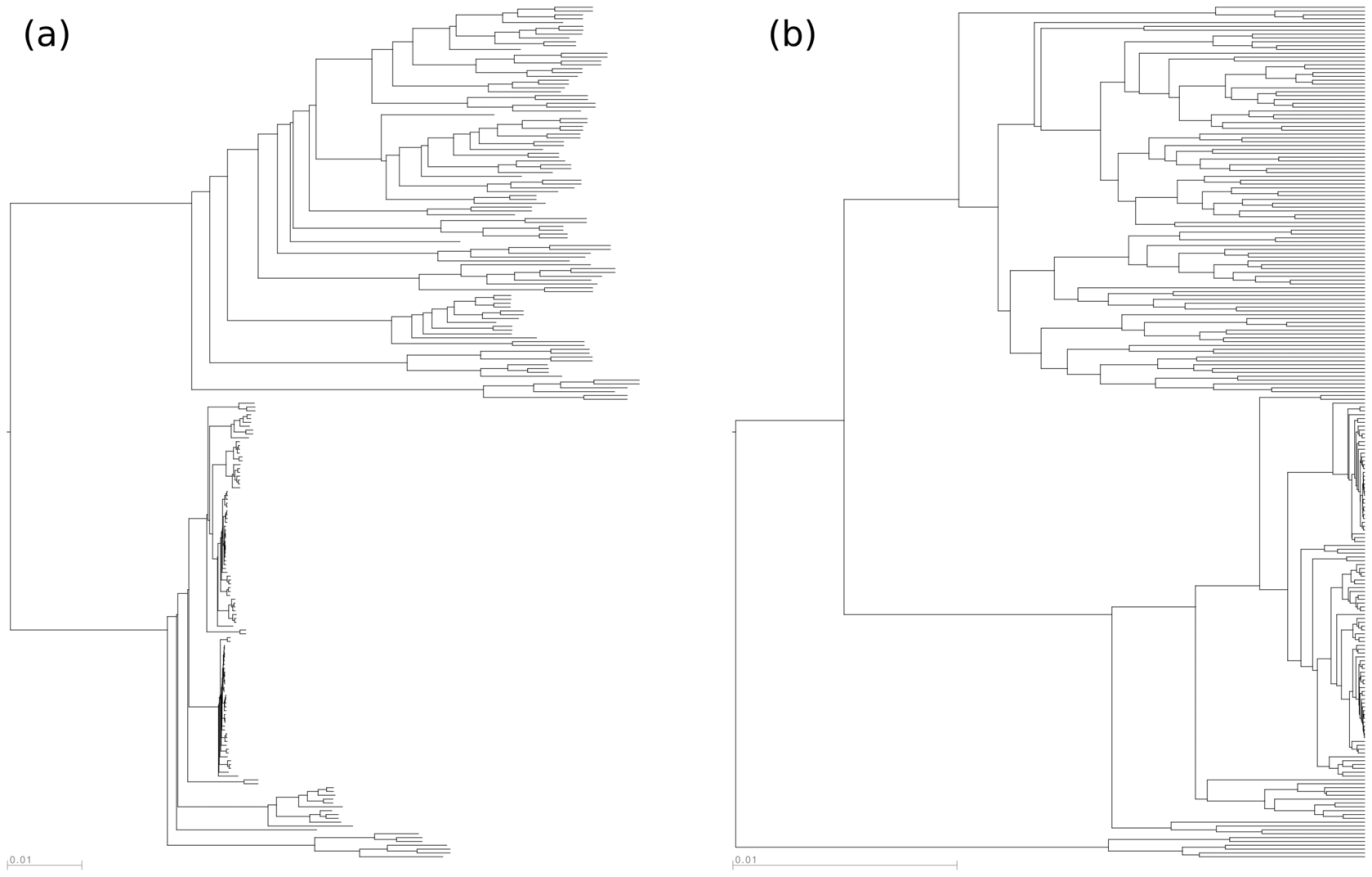
The results of (a) squash clustering and (b) UPGMA as applied to the vaginal data. The labels are not shown and they do not appear in the same order on the two trees. For a comparison of labeled trees, see Supplementary Figure S1.

Squash clustering was applied to the collection of vaginal samples in the Fredricks data set. As we have already remarked, because meaningful internal edge lengths can be assigned to the squash clustering tree, it is not ultrametric, whereas the UPGMA tree is (Fig. 8). The two tight clusters at the bottom of (a) and (b) contain the *Lactobacillus* -dominated vaginal samples seen on the left side of (Fig. 6) and correspond to *L. iners* (upper tight cluster) and *L. crispatus* (lower tight cluster). A more detailed leaf-labeled comparison between the two trees is available in the supplementary material (Fig. S1).

### Squash clustering simulation study

It is difficult to find a collection of microbial communities that have a known hierarchical structure, thus simulation was used to validate the effectiveness of the squash clustering methodology. The simulation process is described in detail in the Methods section, but we highlight several important points here. The primary ingredients for the simulation are a fixed “clustering tree” representing the hierarchical relationship between a set of communities and a “reference tree” of species as above. The simulation generates artificial collections of placements on the reference tree for each leaf of the clustering tree. The success of the clustering algorithms is judged by comparing the original clustering tree to the result of the clustering method applied to the artificial collections of placements. This accuracy comparison is done using the rooted Robinson-Foulds (RF) metric (Methods).

A number of parameters determine the steps in the simulation process. Every internal node of the clustering tree is associated with a “reconstructability” parameter; this parameter determines the level of similarity between descendants of that internal node. In this simulation, the reconstructability parameter is set to a single value for all internal nodes of the tree.

**Figure 9:**
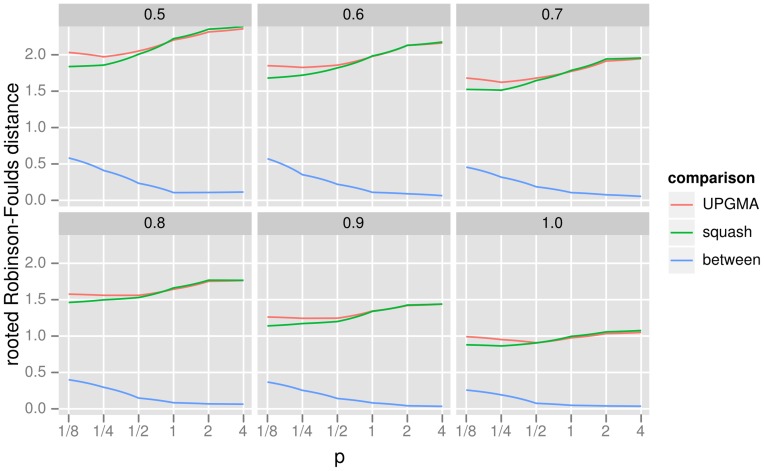
The results of the cluster accuracy simulation experiment using the rooted Robinson-Foulds (RF) metric. This graphic shows very similar levels of topological accuracy for squash clustering and UPGMA, as well as high similarity between the topology returned by the two methods. The figure is divided into panels by the level of reconstructability parameter 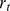 as described in the text (a larger 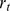 implies easier reconstruction). The 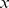-axis is the value of 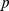 for the 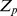 distance as described in (1). The 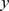-axis is the rooted Robinson-Foulds distance: for the “squash” and “UPGMA” lines it is the distance between the reconstructed tree and the original tree using these two algorithms (lower is more accurate), while the “between” line shows the distance between the result for the two clustering algorithms (lower is more similar). Note that the maximum rooted RF distance between two trees with six taxa is four.

Our simulations show that squash clustering and UPGMA applied to KR distances perform similarly across a wide range of simulation parameters (Fig. 9). Not only do the squash clustering and UPGMA methods have similar levels of accuracy, but their results are also topologically quite similar to one another. Thus squash clustering, with its more transparently meaningful branch lengths, may prove to be an attractive choice for researchers wishing to find hierarchical structure in their data.

## Discussion

### Conclusions

Direct nucleic acid sequencing from environments – ranging from the human body to acid mine drainages – has revolutionized our understanding of the microbial world. In parallel, computational techniques have made great leaps forward in their capacity to classify reads taxonomically [26] and map them onto phylogenetic trees [8], [9]. There has also been a considerable amount of work on useful ways to derive distances between samples [2], [3], [27].

In our paper we have established a new method, “edge principal components analysis” (edge PCA), that associates the principal components axes with signed weightings on the edges of a phylogenetic tree of the species under consideration. By using colors and thickness to visualize these weightings, the user can gain an understanding of what phylogenetic factors drive the separation of the samples. Because the comparison is done in an explicitly phylogenetic context, edge PCA can pick up consistent differences between samples that are subtle from a distance-based standpoint but are readily apparent from the richer tree-based one.

We have also developed a variant of UPGMA, “squash clustering”, that enables visualization of the internal nodes of clustering trees. Because the clustering is done directly on the type of mathematical object that are being visualized, one gains insight into what is driving a particular clustering step.

In this paper we describe these methods and demonstrate their practical effectiveness via an application to vaginal microbiome data. We present simulation results demonstrating the effectiveness of the squash clustering technique in recovering hierarchical structure. In the Methods section, we explain the methods more formally, offer theory connecting these new techniques each other, and show consistency of squash clustering in a simple setting.

In future work we will apply the basic step of the edge principal components method — transforming phylogenetic placement samples into vectors indexed by the edges of the tree — in other contexts. In this paper, we followed this transformation with an application of principal components analysis, but many other options are possible. Our next step will be to apply classical supervised learning techniques to similarly transformed data.

### Generalization and limitations

The methods described here, although implemented for comparison of microbial communities, may in fact be used in more general settings. Edge PCA may be used whenever each sample can be represented by a collection of mass distributed over a common tree structure. Squash clustering may be applied in any case where there is a well-defined notion of the distance between two samples and a well-defined procedure for averaging two samples to produce another object of the same type.

There are some limitations to the sort of comparisons that can be performed using these methods simply because the underlying data is a collection of phylogenetic placements on a tree. For example, if a clade of the reference tree is missing, then differences in read distribution within that clade are not be accounted for in the comparison. Such issues will be present whenever a reference tree is being used, whether using phylogenetic placements directly or mapping reads to the tree using BLAST as a preliminary step in a UniFrac analysis. This disadvantage is balanced by the advantage of not having to define operational taxonomic units (OTU's) by clustering, which can be sensitive to methodological parameters [28].

We also note that the algorithm is influenced by the level of taxon sampling in various regions of the reference tree in such a way that more highly sampled lineages will be assigned comparatively more weight in the PCA analysis than less sampled lineages. This is simply because increasing the level of sampling that produced the reference tree in some region can turn a single edge into multiple edges, and the difference in mass assigned to the single variable that corresponded to the “old” edge is now replicated for each of the variables that correspond to the “new” edges. It can be seen from the variational characterization of the corresponding eigen-problem (see Methods) that the sum of the magnitudes of the eigenvector components corresponding to the “new” edges will be typically greater than the magnitude of the eigenvector component corresponding to the “old” edge. This does not change the interpretation of the location of points relative to the weightings on a tree, however, it does mean that highly sampled lineages may have a disproportionate influence on the construction of the principal components. We are currently developing an alternate formulation that uses mass differences on either side of each point in the reference tree in a manner analogous to the way edge PCA uses mass differences on either side of each edge. The new formulation does not treat all edges as being on an equal footing; rather, it implicitly incorporates information about edge lengths. This “length PCA” procedure will therefore not be perturbed by taxon sampling levels in the same way.

The methods presented here also depend on the number of phylogenetic placements being correlated with the number of organisms of that type found in the sample. This is not always true. Loci such as 16S are often sequenced by first amplifying using a polymerase chain reaction with a broad-spectrum primer; this primer may have different efficiencies for different organisms, or may miss certain organisms altogether. In addition, genetic material extraction efficiency varies by organism [29]. Nevertheless, the results on this example data using our methods do correspond with analyses made with non-genetic methods such as morphological comparison (Fig. 6).

## Methods

### General setting for methods

Phylogenetic placement is a way to analyze the results from high-throughput sequencing applied to DNA extracted in bulk from an environmental sample of microbes. It is simply the assignment of sequencing reads to a “reference” phylogenetic tree constructed from previously-characterized DNA sequences; recent algorithms have focused on doing so according to the phylogenetic maximum-likelihood criterion [8], [9]. By fixing a reference tree rather than attempting to build a phylogenetic tree for the sample from scratch, recent algorithms of this type are able to place tens of thousands of query sequences per hour per processor on a reference tree of one thousand taxa (e.g. species), with performance scaling linearly in the number of reference taxa, the number of query sequences, and the length of the query sequences.

A probability measure on the reference phylogenetic tree is obtained from a collection of sequence reads as follows. A given read can be assigned to the phylogenetic tree in its maximum likelihood or maximum posterior probability location using the phylogenetic likelihood criterion to obtain a “point placement.” A point placement can be thought of as a probability measure with all of the mass concentrated at the best attachment location. Alternatively, one can express uncertainty in the optimal location by spreading the probability mass according to posterior probability (assuming some priors) or “likelihood weight ratio”; see [9] for details. In either case, each read is thought of as a probability measure on the reference phylogenetic tree. A probability measure for a collection of reads can be obtained by averaging the measures for each read individually (that is, by constructing the probability measure that is the mixture of the probability measures for each read in which each such measure is given an equal weight).

### Edge principal components analysis

Begin with a phylogenetic tree 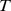 and probability measures 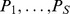 on 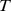, each of which comes from an assignment of the reads in one of 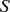 samples to the phylogenetic tree, as described above. If 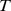 is not already rooted at some vertex, pick an arbitrary vertex to be the root. Removing a given internal edge 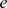 from the tree splits 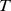 into two components: 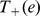 containing the root and 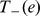 without. For a probability measure 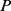 on 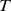, define the corresponding *edge mass difference*

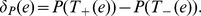
Suppose that 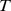 has 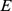 internal edges. The *edge mass difference matrix* 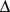 is the 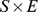 matrix that has the vectors of edge mass differences for the successive samples as its rows. Edge principal components analysis is then performed by first deriving the 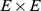 covariance matrix 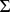 from the matrix 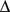 of “observations” followed by computing the 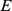 eigenvectors of 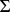 ordered by decreasing size of eigenvalue (Fig. 3).

Each resulting eigenvector is then a signed weighting on the internal edges of the tree, and these weightings may be used to highlight those edges of the tree for which there is substantial between-sample heterogeneity in the masses assigned to the two components of the tree defined by the edge. Indeed, recall the variational characterization of the eigenvectors 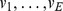 of an 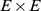 non-negative definite matrix 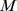 listed in order of decreasing eigenvalue:
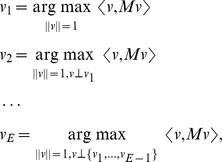
where 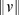 is the usual Euclidean length of the vector 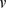, 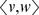 is the usual Euclidean inner product of the vectors 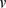 and 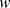, and 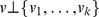 indicates that 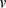 is perpendicular to each of the vectors 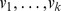. Thus, an edge that receives a weight with large magnitude from an eigenvector corresponding to one of the bigger eigenvalues of the covariance matrix 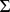 may be viewed as an edge across which there are substantial dissimilarities between samples in the amount of mass placed in the components on either side of the edge.

In our visualization tool, each eigenvector is represented by a single colored and thickened reference tree: the thickness of an edge is proportional to the magnitude of the corresponding entry of the eigenvector and the color specifies the sign of that entry (Fig. 4 and Fig. 5). For the trees shown here, orange signifies a positive entry, while green represents a negative entry. Moreover, we can project each sample onto an eigenvector to visualize how the sample is spread out with respect to that “axis” (Fig. 6).

When considering the weight assigned to a single edge in isolation, only the magnitude of the weight matters and not the sign, because if 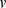 is an eigenvector for a particular eigenvalue, then so is 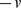. However, sign matters when comparing the weights assigned to two or more edges: if the edge mass differences for two edges are strongly negatively associated, then the corresponding entry of the covariance matrix will be very negative, and the corresponding two entries of the eigenvector for a large eigenvalue will have different signs.

Changing the chosen root from vertex 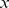 to vertex 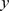 does not affect the eigenvalues or the magnitudes of the entries in the corresponding eigenvectors, and it only changes the signs of the entries for the edges between 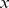 and 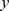. This may be seen as follows. Note first that if an edge 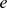 is between 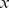 and 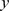, then re-rooting flips the sign of 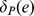, whereas 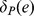 is remains the same if 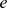 is not between 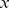 and 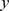. Define 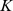 to be the diagonal 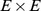 matrix such that 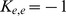 for edges 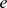 on the path between 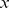 and 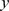, and 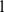 otherwise. Note that 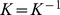. The covariance matrix 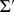 for the re-rooted tree and that for the original tree are related by a similarity transformation: 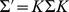. Thus, the eigenvalues for 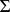 are the same as those for 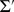, and 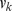 is an eigenvector of 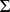 if and only if 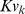 is an eigenvector of 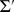.

As with classical principal components analysis, the question arises of choosing an appropriate number 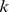 such that the eigenvectors corresponding to the 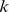 largest eigenvalues represent “signal” in the data, whereas the remaining 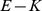 eigenvectors represent “noise”. That is, one wishes to choose 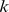 such that the projection of the data onto the subspace spanned by the first 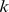 eigenvectors is a reasonably faithful lower-dimensional summary of the data that does not miss important features. There is no clear-cut, “one-size-fits-all” solution to this problem. The usual approach is to first construct a *scree plot* that depicts for each  the proportion of the total variance explained by the 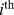 eigenvector (that is, 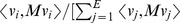] and the proportion of the total variance explained by the first 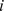 eigenvectors (that is, 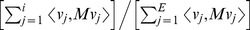). One then chooses 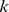 so that the there is a substantial jump from the proportion of variance explained by the 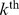 eigenvector to the proportion explained by the 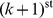 and, moreover, so that the proportion of variance explained by the first 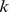 eigenvectors is close to 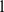. Also, a wish to represent the data graphically by plotting the projection onto the subspace spanned by the first 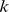 eigenvectors makes a choice of 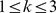 desirable if it is reasonable in terms of the above criteria.

We now shift our attention to support overlap minimization. We will measure overlap of vectors 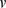 and 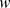 two ways: either in an 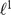 sense by considering 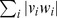, or in an 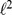 sense by considering 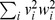. Either of these can be extended to define an overlap of a collection of vectors by considering the sum of their pairwise overlaps.

The rotation idea is simple: rotate the eigenvectors in the space that they span. Specifically, assume that we want to apply this process to the first 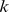 eigenvectors; let 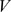 be the matrix with the first 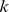 eigenvectors as columns. Such a rotation can be obtained by multiplying 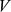 on the right by an arbitrary 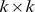 rotation matrix 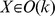; the columns of the resulting matrix are the rotated eigenvectors. The rotation Support Overlap Minimization (SOM) process finds the rotation that minimizes the 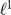 overlap function applied to 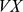 for 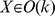.

One disadvantage of the rotation process is that the axes lose their inherent meaning; for example, the first dimension is no longer the axis of maximal variance. An alternative means of minimizing support overlap is to explicitly penalize the 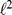 overlap. For the second component, this can be done by taking the highest-eigenvalue eigenvector of the matrix 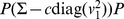, where P is the projection onto the orthogonal complement of the span of 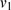, which can be obtained by power iteration. In that case,
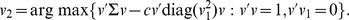
We have not been as successful with this approach as with the rotation described above; in the examples we have tried a large value of 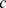 is needed to see a significant decrease in overlap, but that leads to an excessive distortion of the principal component vectors.

### Squash clustering

Squash clustering is a type of hierarchical clustering using the earth-movers, or Kantorovich-Rubinstein (KR) distance described above. The key difference with other types of hierarchical clustering happens when merging two clusters: we simply take a weighted average of the two corresponding mass distributions to get the mass distribution that corresponds to the new, larger cluster (Fig. 2).

Agglomerative hierarchical clustering in general proceeds by iterating the following sequence of steps until there is a single cluster and a corresponding 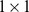 pairwise distance matrix.

1. Find the smallest off-diagonal element in the current pairwise distance matrix. Say it is the distance between clusters 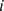 and 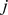.
2. Merge the 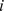 and 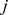 clusters, making a cluster 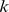.
3. Remove the 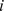
th and 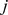
th rows and columns from the distance matrix.
4. Calculate the distance from the cluster 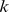 to the other clusters.
5. Insert the distances from 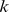 into the distance matrix.

Classical hierarchical clustering methods calculate the distance in step number 4 as some function of the distance matrix. In particular, average-linkage clustering or UPGMA calculates the distance between two clusters as the average between pairs of items in the clusters.

Squash clustering takes the average of the mass distributions and then computes KR distances from the merged cluster to the other clusters. That is, if we merge two clusters that correspond to sets of 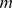 and 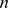 original mass distributions and are represented by averaged mass distributions 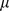 and 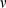, then the new cluster is represented by the mass distribution
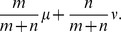
Because these merged clusters are simply mass distributions, one can calculate KR distances as usual for the next stage of clustering. The series of merges in the clustering algorithm determines the topology of the rooted *clustering tree* that the algorithm produces. Leaves of the tree correspond to individual samples.

The KR distances between these mass distributions can be used to assign branch lengths to the clustering tree. Specifically, each internal node is associated with exactly one mass distribution, and the length of a given branch between two internal nodes 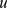 and 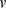 is equal to the KR distance between the mass distributions associated with 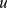 and 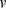. The mass distributions corresponding to the internal nodes of the phylogenetic tree can be visualized using the software implementation. In contrast, for UPGMA the branch lengths are differences of “heights” that are calculated as certain averages of distances from the original distance matrix. (We note that in the default UPGMA implementation in R, the branch lengths for “pendant” branches leading to leaves are arbitrarily specified by the user and thus the trees may not appear ultrametric.)

In the next section, we investigate connections between edge PCA and squash clustering, compare squash clustering and UPGMA in more detail, and show that squash clustering is consistent given ultrametric data.

### Further results

Given probability measures 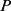 and 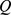 on the rooted tree 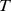, the Zolatarev-like 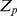 generalization of the KR distance is defined for 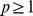 as
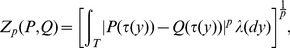
(1)where 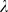 is the natural length measure on the tree and 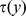 is the subtree on the other side of 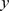 from the root [7]. The classical KR distance is (1) with 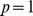; this is the value that corresponds to weighted UniFrac. It is shown in [7] that choosing a different root does not change the distance. It is also noted there that if 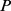 and 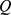 only assign mass to leaves of the tree and 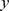 is in the interior of edge 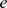 then
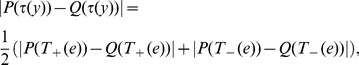
furnishing a connection with edge PCA.

At each stage of the squash clustering algorithm we have a pairwise distance matrix with rows and columns indexed by the clusters that have already been made by the algorithm. Initially, the clusters are just the individual samples and the entries in the pairwise distance matrix are computed using equation (1).

We now compare UPGMA and squash clustering in more detail. For UPGMA, if clusters 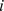 and 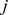 containing respective numbers of items 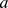 and 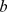 are merged to form a cluster 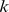 with 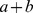 items, then the average-linkage distance between another cluster 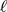 with 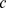 items and the new cluster 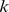 is (writing 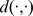 for the distance between individual items)
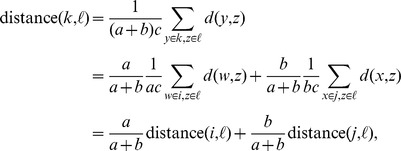
and so the entries of the updated UPGMA distance matrix are just suitably weighted averages of the entries of the previous distance matrix.

At each stage of squash clustering, on the other hand, a cluster is associated with a probability measure on the tree 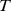. When two clusters 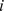 and 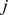 containing respective numbers of items 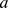 and 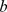 and associated with respective probability measures 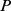 and 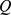 are merged to form a cluster 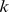, then the new cluster 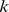 is associated with the probability measure 
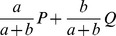
 and the distance from 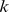 to some other cluster 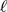 associated with the probability measure 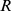 is
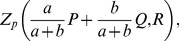
(2)which is analogous to the above equation for the UPGMA averaging procedure. As remarked above, the “squash” interpretation of (2) comes from recalling that the probability measures associated with the two clusters are each simple averages of all of the measures for the items in the clusters (Fig. 2). That is, if 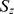 is the probability measure associated with original item 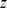, then
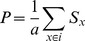
and
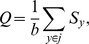
and the probability measure associated with the new cluster 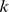 is
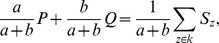
the (unweighted) average of the probability measures in 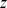.

A natural question to ask is whether the distance between a probability measure 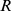 and the weighted average of two probability measures 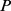 and 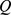 is equal to the similarly weighted average of the distance between 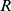 and 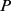 and the distance between 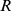 and 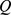. The answer is in general “no”: starting from (1) we have from the Minkowski inequality that for 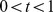:
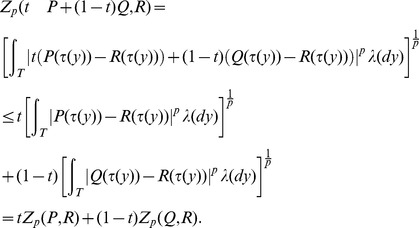
The early iterations of the UPGMA and squash clustering algorithms can be quite similar because the pairs of objects being merged are close together relative to their distance to the other objects. For example, if 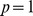, then the above inequality is an equality whenever 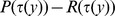 and 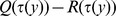 have the same sign for all 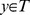.

#### Consistency of squash clustering on ultrametric data

An appealing feature of UPGMA is that if the pairwise distances which are used to initialize the algorithm are the leaf-to-leaf distances for an ultrametric rooted tree 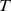, then UPGMA is guaranteed to return 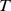. In this section we show that squash clustering has a similar property in a simple special case. This observation complements the validation work done using simulation to show that squash clustering does recover hierarchical structure when it is present.

In order to explain the result for squash clustering, we must first review the simple demonstration of the above result for UPGMA.

Imagine that the ultrametric rooted tree 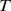 is oriented on the page with the root at the top and the leaves at the bottom, and for simplicity assume that it is a bifurcating tree. By the assumption of ultrametricity, all the leaves will sit on a horizontal line. Imagine the internal nodes 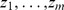 of 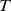 are listed in order of increasing distance from the line so that 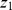 is the closest. For simplicity, suppose further that no two of these distances are equal, so that we don't have to adopt an arbitrary convention for breaking ties. Each internal node corresponds to a set of leaves – namely, those that are below it.

We proceed inductively to demonstrate that the merges done by the algorithm reproduce, in order, the sets of leaves below the internal nodes 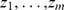 and that the distances between clusters assigned by UPGMA agree with the original node-to-node distances in 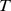. The base case is trivial. Assume the algorithm satisfies the inductive hypothesis for all 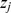 with 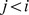. The two nodes descending from 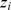 in 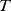 are each an internal node of the form 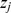 for some 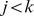 or a single leaf. Call the two corresponding sets of leaves below these nodes 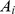 and 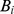. By induction, 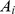 and 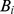 are present among the clusters that have been constructed by UPGMA after the 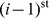 merge. The distance in 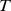 between any pair of leaves 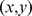 with 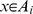 and 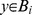 is the same. By construction, the UPGMA distance between 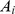 and 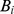,
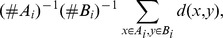
is equal to the distance between any two such leaves 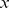 and 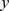. Furthermore, the UPGMA distance between 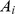 (resp. 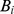) and any other cluster 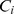 present after 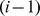 UPGMA merges is equal to the common distance in 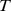 between any leaf in 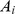 (resp. 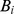) and any leaf in 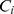. Moreover, by the definition of 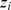, this common distance is greater than the UPGMA distance between the clusters 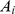 and 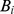. It is now clear that the 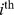 merge of UPGMA merges the clusters 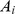 and 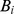 to produce a cluster that coincides with the set of leaves below 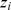 in 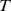 and that the updating of distances maintains the agreement between node-to-node distances in 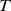 and UPGMA cluster-to-cluster distances.

A similar argument leads to an analogous statement for squash clustering. Again, assume that the reference tree 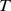 is an ultrametric rooted tree. For each leaf 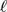, assume that there is a single sample 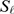 consisting of a single read mapped to 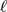. We will show that in this case both squash clustering and UPGMA applied to KR 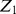 distances return the reference tree 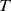 as the clustering tree.

First note that the 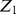 distance between the two samples 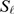 and 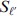 is simply the distance on the tree between the leaves 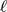 and 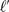. These distances are ultrametric by assumption. Thus, UPGMA run with KR distances will return 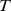 as the clustering tree in this case.

Further, squash clustering and UPGMA start with the same clusters (each read in a cluster by itself), every cluster is trivially the set of leaves below a node of the reference tree 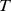, and the distances between clusters are the same for the two methods. Suppose, then, that after some number of iterations of the two methods we are still in a situation where the two methods have the same clusters available to merge, these clusters are disjoint sets of leaves below nodes of 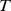, and the distances between the clusters available to merge are the same for the two methods.

Call the available clusters 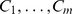. By definition, squash clustering and UPGMA will merge the same pair of clusters – say, without loss of generality, 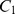 and 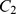. The 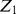 squash clustering distance is the optimal transport (earth movers') distance between the probability measure that puts mass 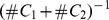 at each leaf of 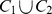 and the probability measure that puts mass 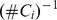 at each leaf of 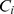 for 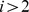. Because, as we remarked above, 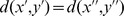 for any 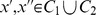 and 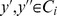, 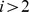, the optimal transport distance is necessarily this common value. Thus, the updating of the distances between the clusters available for merging is the same for the two methods. Therefore, by induction, the trees produced by the two methods will be the same and will coincide with the tree 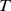.

### Simulation methodology for clustering validation

In this section we present methodology for making artificial “samples” that are hierarchically related. These are then used to compare squash clustering to UPGMA. The code for these simulations can be found on the commiesim branch of pplacer at http://github.com/matsen/pplacer/tree/commiesim.

Start with a true “clustering tree” 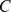: the tree of communities on which we are simulating. Let 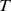 be a phylogenetic “reference” tree of the organisms of interest: the phylogenetic tree of the actual species from which the simulated placements will be drawn. Write 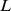 for the set of leaves of 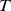. Before describing the simulation we recall some standard terminology. A *split* of 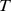 is the partition of the leaves 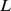 induced by an edge of 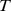: it consists of the two subsets of 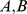 of 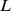 that are on either side of the edge. We have 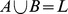 and 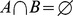, and we use the notation 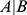 to denote that the subsets 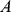 and 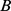 form a split.

The first step of simulation assigns subsets of 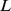 to the leaves of the clustering tree 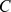. The elements of each such subset are the organisms found in that particular “community”; the community will then be used to generate simulated placements by sampling some number of members of the community with replacement. For example, suppose that a leaf 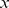 of the clustering tree 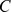 is associated with the set 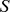 of leaves of the reference tree 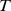; to generate a sampled collection of placements for 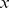 we first sample from 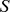 with replacement. The resulting multi-set of leaves of the reference tree 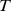 is made into a collection of placements by turning each element into a placement consisting of a unit point mass at the given leaf of the reference tree.

These simulated collections of placements are then used to reconstruct the clustering tree by applying either squash clustering or UPGMA on the KR distances.

Subsets of the leaf set 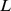 of 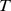 are assigned to leaves of the clustering tree 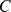 by a recursive procedure that proceeds down the clustering tree beginning with the root 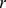. At each stage there is a current internal node 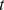 of 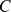 and a set of leaf sets 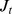 associated with 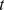. The recursion is initialized with 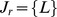. We proceed down the tree 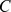 from a node 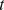 in two stages: we first split the set of subsets 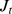 and then assign some of these subsets to each child of 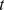.

The splitting stage is done by selecting splits (a.k.a. bipartitions) of 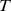 and using them to cut apart the leaf subsets. For example, suppose that 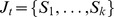 is the set of subsets of 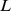 associated with the internal node 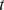 of 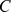 that we are currently processing. We select an “effective” split 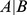 of 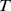 i.e. one such that 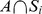 and 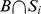 are non-empty for some 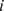. Applying this split produces the new collection of leaf subsets 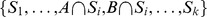. Each one of the 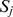 corresponds to a connected region of the reference tree 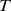, and applying an effective split corresponds to disconnecting one of those regions by cutting an edge of 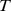. In the simulation, we sample an integer 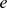 from a Poisson distribution with mean 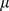 and then sample 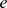 effective splits uniformly with replacement from the set of all effective splits for the subsets in 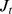. We apply those splits successively as above to split the subsets in 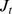. This splitting produces a new set of leaf subsets that we call 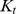.

Next, for each child of the current internal node 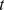, we select a subset of 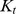 of size 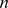 to pass on to the child. We do this in such a way that 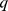 of the subsets selected are the same for each child, while the remaining 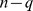 are selected independently of the corresponding selections for the other children. Here 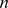 is a fixed parameter and 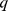 is a realization of a binomial distribution with number of trials 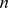 and success parameter 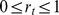. The “reconstructability parameter” 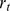 determines the level of similarity between the children of 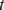: for internal nodes with high 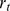 the subsets assigned to its children will be quite similar, while for those with low 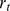 the subsets will tend to be different.

More specifically, suppose that the children of 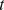 are the nodes 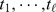. We first sample 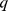 elements from 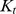 with replacement to make a set 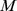 of subsets of 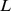 with at most 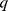 elements. Next, for 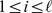, we sample 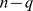 elements from 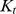 with replacement to make a set 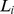 of subsets of 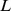 with at most 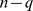 elements. Then, 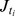, the set of subsets associated with the node 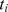, is defined to be the set 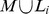. By recurring in this fashion, every node 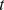 of the clustering tree 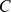 is assigned some set 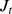 of subsets of the set of leaves 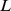 of the reference tree 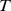. For each leaf 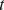 of the clustering tree, placements are simulated as described above from the set of leaf subsets 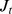.

For the study reported in Figure 9, the following parameters were used. The clustering tree 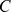 was, in the usual “Newick” bracketing notation for binary rooted trees, the tree 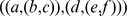. The reference tree 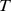 was the tree for microbes in the vaginal environment used in the rest of the paper. 500 trials were performed for every parameter setting, and 100 placements were generated for each clustering leaf of each trial. The mean number of cuts 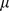 was set to 10, and the number of sets selected 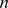 was set to 5. The reconstructability parameters 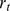 for all internal nodes were set to the value specified in the panel label of the figure.

The Robinson-Foulds (RF) metric [30] of two trees 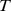 and 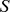 was computed as half the size of the symmetric difference of the split-set of 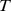 and that of 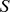. Because the classical RF distance is calculated on unrooted trees, while the clustering trees in the study are rooted, we attached a fictitious “root leaf” to the root before calculating RF distances to account for the position of the root. We call the resulting quantity the *rooted Robinson-Foulds distance*. For a bifurcating tree on six leaves such as 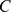, the maximal rooted RF distance is four.

## Supporting Information

Figure S1
**A comparison of the clustering results for the Fredricks data using the software of** [**32**] **.** The software uses the Hungarian (a.k.a. Munkres) algorithm to find an optimal one-to-one matching between edges of the trees minimizing differences in a topological score between pairs of matched branches as follows. Given two trees 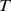 and 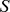 on the same samples, let 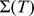 and 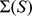 be the bipartitions of the samples induced by cutting the edges of 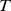 and 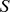. For two bipartitions 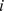 and 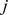, one associates an “agreement score” 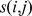 describing the proportion of shared elements between the sides of the bipartitions. The algorithm finds a one-to-one matching between 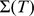 and 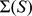 that minimizes the total agreement score between matched bipartitions. Each tree is drawn in a way which shows the agreement scores: a thick branch represents an edge which has a low agreement score with its partner in the matching. The program arranges the trees such that matched edges are close to one another on the tree. Branches shown in red mean the colored branch is longer than the branch in the other tree, while those in blue are opposite; the intensity of the color indicates the degree of this difference.(PDF)

Figure S2 **The combined vaginal samples divided by race, plotted with respect to the first two principal components and colored by Nugent score.**
(TIFF)

## References

[1] Jaccard P (1908) Nouvelles recherches sur la distribution orale. Bull Soc Vaudoise Sci Nat 44: 223–270.

[2] Lozupone C, Knight R (2005) UniFrac: a new phylogenetic method for comparing microbial communities. Appl Environ Microbiol 71: 8228. 16332807 10.1128/AEM.71.12.8228-8235.2005 PMC1317376

[3] Lozupone CA, Hamady M, Kelley ST, Knight R (2007) Quantitative and qualitative beta diversity measures lead to different insights into factors that structure microbial communities. Appl Environ Microbiol 73: 1576–85. 17220268 10.1128/AEM.01996-06 PMC1828774

[4] Costello E, Lauber C, Hamady M, Fierer N, Gordon J, et al (2009) Bacterial community variation in human body habitats across space and time. Science 326: 1694–1697. 19892944 10.1126/science.1177486 PMC3602444

[5] Ley R, Turnbaugh P, Klein S, Gordon J (2006) Microbial ecology: human gut microbes associated with obesity. Nature 444: 1022–1023. 17183309 10.1038/4441022a

[6] Nemergut D, Costello E, Hamady M, Lozupone C, Jiang L, et al (2011) Global patterns in the biogeography of bacterial taxa. Environ Microbiol 13: 135–144. 21199253 10.1111/j.1462-2920.2010.02315.x PMC5834236

[7] Evans SN, Matsen FA (2012) The phylogenetic Kantorovich-Rubinstein metric for environmental sequence samples. J Royal Stat Soc (B) 74: 569–592. 10.1111/j.1467-9868.2011.01018.x PMC3405733 22844205

[8] Berger SA, Krompass D, Stamatakis A (2011) Performance, accuracy, and web server for evolutionary placement of short sequence reads under maximum likelihood. Syst Biol 60: 291. 21436105 10.1093/sysbio/syr010 PMC3078422

[9] Matsen FA, Kodner RB, Armbrust EV (2010) pplacer: linear time maximum-likelihood and Bayesian phylogenetic placement of sequences onto a fixed reference tree. BMC Bioinformatics 11: 538. 21034504 10.1186/1471-2105-11-538 PMC3098090

[10] Wang H, Marron J (2007) Object oriented data analysis: Sets of trees. Ann Stat 35: 1849–1873.

[11] Nye T (2011) Principal components analysis in the space of phylogenetic trees. Ann Stat 39: 2716–2739.

[12] Bik EM, Eckburg PB, Gill SR, Nelson KE, Purdom EA, et al (2006) Molecular analysis of the bacterial microbiota in the human stomach. Proc Natl Acad Sci USA 103: 732–7. 16407106 10.1073/pnas.0506655103 PMC1334644

[13] Purdom E (2008) Analyzing data with graphs: Metagenomic data and the phylogenetic tree. UC Berkeley Statistics Technical Reports 766: 1–22.

[14] Mitra S, Klar B, Huson DH (2009) Visual and statistical comparison of metagenomes. Bioinformatics 25: 1849–55. 19515961 10.1093/bioinformatics/btp341

[15] Segata N, Izard J, Waldron L, Gevers D, Miropolsky L, et al (2011) Metagenomic biomarker discovery and explanation. Genome Biol 12: R60. 21702898 10.1186/gb-2011-12-6-r60 PMC3218848

[16] Matsen F, Hoffman N, Gallagher A, Stamatakis A (2012) A format for phylogenetic placements. PLOS ONE 7: e31009. 22383988 10.1371/journal.pone.0031009 PMC3284489

[17] Srinivasan S, Hoffman N, Morgan M, Matsen F, Fiedler T, et al (2012) Bacterial communities in women with bacterial vaginosis: high resolution phylogenetic analyses reveal relationships of microbiota to clinical criteria. PLoS ONE 7: e37818. 22719852 10.1371/journal.pone.0037818 PMC3377712

[18] Gentleman RC, Carey VJ, Bates DM, Bolstad B, Dettling M, et al (2004) Bioconductor: Open software development for computational biology and bioinformatics. Genome Biol 5: R80. 15461798 10.1186/gb-2004-5-10-r80 PMC545600

[19] R Development Core Team (2011) R: A Language and Environment for Statistical Computing. R Foundation for Statistical Computing, Vienna, Austria. Available: http://www.R-project.org.

[20] Ravel J, Gajer P, Abdo Z, Schneider G, Koenig S, et al (2011) Vaginal microbiome of reproductive-age women. Proc Natl Acad Sci USA 108: 4680. 20534435 10.1073/pnas.1002611107 PMC3063603

[21] Cole JR, Wang Q, Cardenas E, Fish J, Chai B, et al (2009) The Ribosomal Database Project: improved alignments and new tools for rRNA analysis. Nucleic Acids Res 37: D141–D145. 19004872 10.1093/nar/gkn879 PMC2686447

[22] Stamatakis A (2006) RAxML-VI-HPC: maximum likelihood-based phylogenetic analyses with thousands of taxa and mixed models. Bioinformatics 22: 2688–2690. 16928733 10.1093/bioinformatics/btl446

[23] Nawrocki E, Kolbe D, Eddy S (2009) Infernal 1.0: inference of RNA alignments. Bioinformatics 25: 1335–1337. 19307242 10.1093/bioinformatics/btp157 PMC2732312

[24] Nugent RP, Krohn MA, Hillier SL (1991) Reliability of diagnosing bacterial vaginosis is improved by a standardized method of gram stain interpretation. J Clin Microbiol 29: 297–301. 1706728 10.1128/jcm.29.2.297-301.1991 PMC269757

[25] Zozaya-Hinchliffe M, Martin D, Ferris M (2008) Prevalence and abundance of uncultivated megasphaera-like bacteria in the human vaginal environment. Appl Environ Microbiol 74: 1656. 18203860 10.1128/AEM.02127-07 PMC2258643

[26] Brady A, Salzberg S (2009) Phymm and PhymmBL: metagenomic phylogenetic classification with interpolated markov models. Nature methods 6: 673–676. 19648916 10.1038/nmeth.1358 PMC2762791

[27] Kuczynski J, Liu Z, Lozupone C, McDonald D, Fierer N, et al (2010) Microbial community re- semblance methods differ in their ability to detect biologically relevant patterns. Nature methods 7: 813–819. 20818378 10.1038/nmeth.1499 PMC2948603

[28] White JR, Navlakha S, Nagarajan N, Ghodsi MR, Kingsford C, et al (2010) Alignment and clustering of phylogenetic markers - implications for microbial diversity studies. BMC Bioinformatics 11. 10.1186/1471-2105-11-152 PMC2859756 20334679

[29] Morgan JL, Darling AE, Eisen JA (2010) Metagenomic Sequencing of an In Vitro-Simulated Mi- crobial Community. PLoS ONE 5. 10.1371/journal.pone.0010209 PMC2855710 20419134

[30] Robinson DF, Foulds LR (1981) Comparison of phylogenetic trees. Math Biosci 53: 131–147.

[31] Wickham H (2009) ggplot2: elegant graphics for data analysis, volume 35 of use R! New York: Springer, 217 pp. doi:10.1007/978-0-387-98141-3.

[32] Nye TMW, Liò P, Gilks WR (2006) A novel algorithm and web-based tool for comparing two alternative phylogenetic trees. Bioinformatics 22: 117–9. 16234319 10.1093/bioinformatics/bti720

