# Correction: Edge Principal Components and Squash Clustering: Using the Special Structure of Phylogenetic Placement Data for Sample Comparison

**DOI:** 10.1371/annotation/40cb3123-845a-43e7-b4c0-9fb00b6e2212

**Published:** 2013-06-21

**Authors:** Frederick A. Matsen IV, Steven N. Evans

Images for Figures 2 and 3 were incorrectly switched. The image labeled as Figure 2 is Figure 3, and the image labeled as Figure 3 is Figure 2. 

