# Supplementary figures and images for: Edge Principal Components and Squash Clustering: Using the Special Structure of Phylogenetic Placement Data for Sample Comparison
Source: PLoS One. 2013 Mar 11;8(3):e56859. doi: 10.1371/journal.pone.0056859 (PMC3594297; doi:10.1371/journal.pone.0056859)

squash tree

UPGMA tree

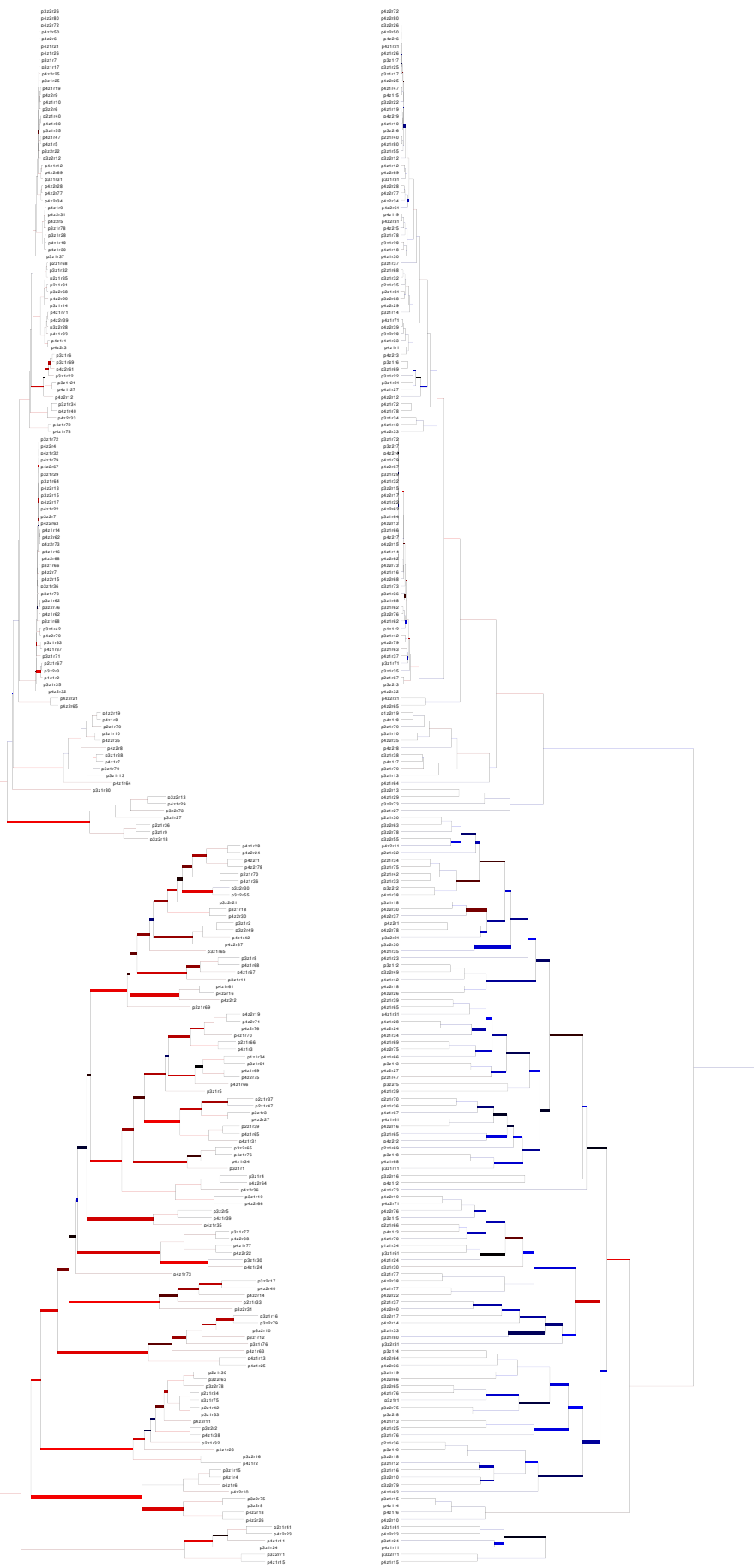

Supplement: Figure S1 — A comparison of the clustering results for the Fredricks data using the software of [32] . The software uses the Hungarian (a.k.a. Munkres) algorithm to find an optimal one-to-one matching between edges of the trees minimizing differences in a topological score between pairs of matched branches as follows. Given two trees and on the same samples, let and be the bipartitions of the samples induced by cutting the edges of and . For two bipartitions and , one associates an “agreement score” describing the proportion of shared elements between the sides of the bipartitions. The algorithm finds a one-to-one matching between and that minimizes the total agreement score between matched bipartitions. Each tree is drawn in a way which shows the agreement scores: a thick branch represents an edge which has a low agreement score with its partner in the matching. The program arranges the trees such that matched edges are close to one another on the tree. Branches shown in red mean the colored branch is longer than the branch in the other tree, while those in blue are opposite; the intensity of the color indicates the degree of this difference. (PDF) [file pone.0056859.s001.pdf]

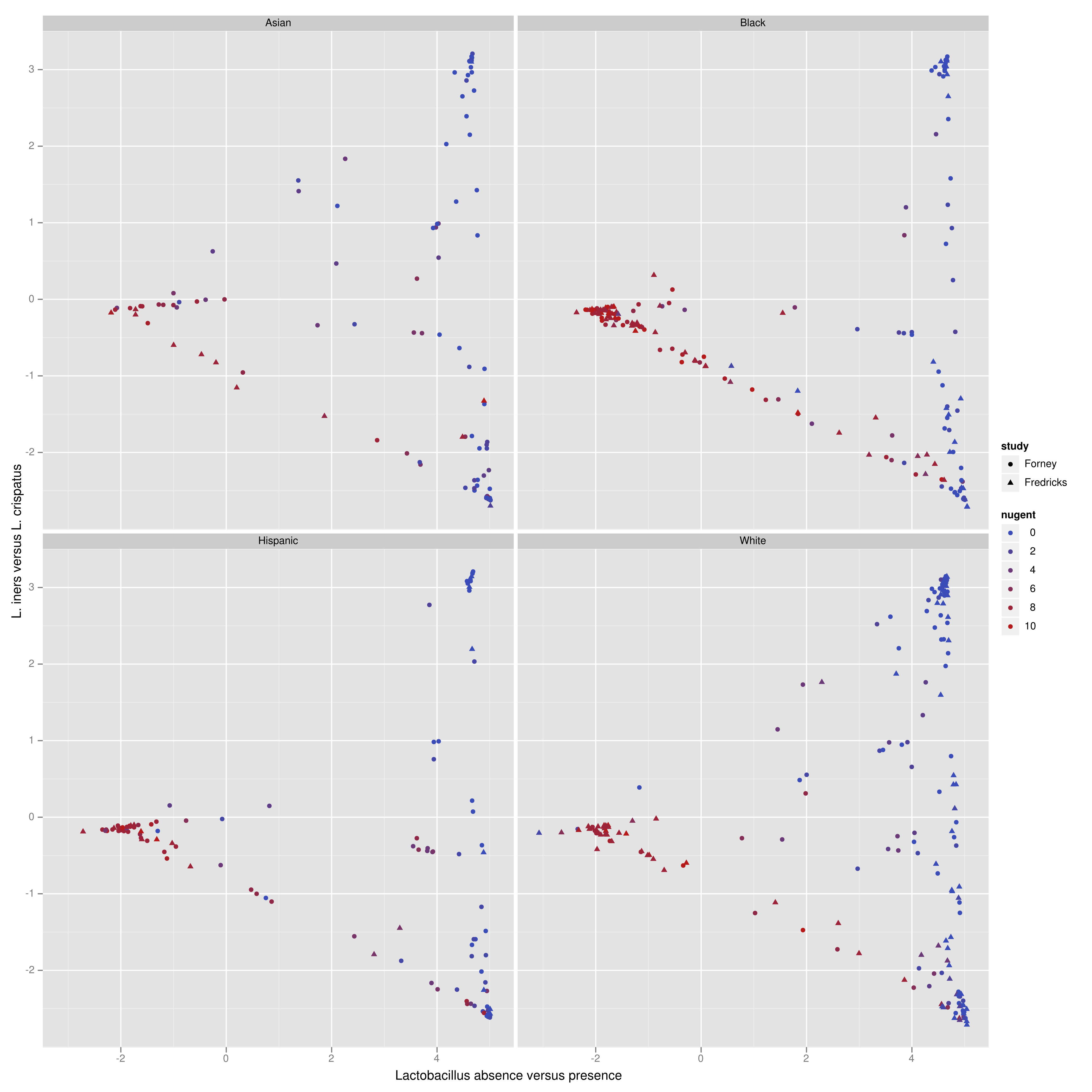

Supplement: Figure S2 — The combined vaginal samples divided by race, plotted with respect to the first two principal components and colored by Nugent score. (TIFF) [file pone.0056859.s002.tiff]
